# Relationships between Sleep Problems and Psychiatric Comorbidities among China's Wenchuan Earthquake Survivors Remaining in Temporary Housing Camps

**DOI:** 10.3389/fpsyg.2016.01552

**Published:** 2016-10-18

**Authors:** Suo Jiang, Zheng Yan, Pan Jing, Changjin Li, Tiansheng Zheng, Jincai He

**Affiliations:** ^1^Institute of Developmental Psychology, School of Psychology, Beijing Normal UniversityBeijing, China; ^2^Department of Applied Psychology, Wenzhou Medical UniversityWenzhou, China; ^3^Department of Educational and Counseling Psychology, University at Albany/State University of New YorkAlbany, NY, USA; ^4^Department of Psychiatry, Ningbo Kangning HospitalNingbo, China; ^5^Department of Neurology, The First Affiliated Hospital of Wenzhou Medical UniversityWenzhou, China

**Keywords:** anxiety, depression, earthquake survivors, PTSD, sleep problems

## Abstract

Earthquake survivors are a diverse population. This study focused on a special group of earthquake survivors, who had still stayed in temporary housing camps for about 2 years after China's Wenchuan Earthquake rather than those who moved back to rebuild their lives or immigrated to large cities to seek new lives. The research goals were to (1) assess their sleep problems as well as their PTSD, depression and anxiety and (2) examine the relationship between different dimensions of sleep quality and PTSD, depression, and anxiety among these survivors. Three-hundred and eighty seven earthquake survivors who remained in temporary housing camps and had sleep problems were recruited 17–27 months after Wenchuan Earthquake. Four standardized instruments-The Pittsburgh Sleep Quality Index, PTSD Checklist-Civilian Version, Self-rating Depression Scale, Self-rating Anxiety Scale, and face-to-face one-on-one structured interviews were used to assess these survivors' sleep quality, PTSD, depression, and anxiety. It was found that (1) 83.20% of these survivors reported having sleep problems, and 79.33% of them considered insomnia as the most common sleep problem; (2) 12.14% suffered PTSD, 36.43% experienced depression, and 38.24% had anxiety; (3) sleep disturbance, sleep medication use, and subjective sleep quality were significantly related to PTSD; (4) habitual sleep efficiency, sleep disturbance, sleep medication use, and daytime dysfunction were significantly related to depression; and (5) sleep disturbance, sleep medication use, and daytime dysfunction were significantly related to anxiety. Clinic implications of the study are discussed.

## Introduction

Earthquakes, as one of the major natural disasters, have strongly associated with various problems of psychiatric morbidity, such as posttraumatic stress disorder (PTSD), depression, and anxiety among Earthquake survivors (Maj et al., 1989; Wood et al., 1992; Kato et al., 1996; Krakow et al., 2000; Chen et al., 2007; Chung et al., 2010; George et al., 2012). The prevalence rate of PTSD after earthquakes varied from 14.5 to 74.0% (Goenjian, 1993; Hsu et al., 2002; Başoglu et al., 2004; Tural et al., 2004; Altindag et al., 2005; Lee et al., 2009; Giannopoulou et al., 2006; Kuo et al., 2007; Priebe et al., 2009). There is also an increasing literature regarding comorbidity of anxiety and depression in children and adolescents following earthquakes (Angold and Costello, 1992; Goenjian et al., 1995). Depressive disorders have been found to be associated with the co-occurrence of anxiety and conduct disorders. Symptoms of both depression and anxiety have been noted to be more severe when these conditions co-occur (Strauss et al., 1988). And there is evidence that psychological distress persists following a disaster (Bland et al., 1996, 2005; Carr et al., 1997; Salcioglu et al., 2003; van Griensven et al., 2006; Hollifield et al., 2008).

On May 12, 2008, the Wenchuan region of China was hit by a particularly destructive earthquake that measured 8.0 on the Richter scale, and 69,227 people were killed, 374,643 injured, 17,923 listed as missing, and 4.8 million left homeless (Wang et al., 2011a; Ma et al., 2013). It has been well-documented that survivors of the Wenchuan earthquake have experienced various problems of psychiatric morbidities. The prevalence rates of probable PTSD in two communities 3 months after the Wenchuan earthquake were 37.8 and 13.0% respectively (Wang et al., 2009). The prevalence rates of the problems at two time-points (6 months and 1 year after the Wenchuan earthquake) were 23.3 and 22.7% for anxiety, 14.5 and 16.1% for depression, and 11.2 and 13.4% for PTSD, respectively, among 330 grades 3–5 children (Liu et al., 2010). The prevalence rates of probable PTSD, anxiety and depression were 26.3, 49.8, and 49.6%, respectively, among hard-hit survivors 1 year after the Wenchuan earthquake (Zhang et al., 2011). Most of the survivors who remained in the community 1 year after the earthquake had scored higher on self-report measures reflecting distressing symptoms and impairment as compared with their departed neighbors, and had a higher prevalence of depression and suicide risk (Ma et al., 2013).

Besides various problems of psychiatric morbidities, earthquake survivors have been usually found to be associated with various types of sleep disorders with varying prevalent rates from 10.7 to 70.0% (North et al., 1999; Johnson et al., 2006; Varela et al., 2008). Earthquakes resulted in increased frequencies of nightmares among survivors (Wood et al., 1992). Given the well-documented impacts of earthquakes on both psychiatric problems and sleep disorders, researchers started to specify the prevalence of sleep problems while examining PTSD, depression, and anxiety (Ohayon and Shapiro, 2000; Spoormaker and Montgomery, 2008; Belleville et al., 2009). Sleep problems commonly co-occur with anxiety disorders broadly and with PTSD more specifically (Babson et al., 2011; Lauterbach et al., 2011) Depression is considered as one of the most prominent causes of insomnia. More than 90% of depressed patients complain about difficulties in falling asleep, frequent nocturnal and early morning awakenings (Berger et al., 2003). Sleep disturbances were often viewed as a secondary symptom of PTSD. However, recently a growing body of evidence show that disturbed sleep is more than a secondary symptom of PTSD but rather a core feature of PTSD (Spoormaker and Montgomery, 2008; Suvak et al., 2008; Gargurevich et al., 2009; Wang et al., 2011b). Wang et al. (2011b) examined the factor structure of PTSD in a large sample of individuals from China and indicated that a four-factor structure emerged, which took sleep disturbance as an independent factor. Suvak et al. (2008) and Gargurevich et al. (2009) also established sleep disorders in their four-factor model of PTSD. More broadly, sufficient and restoring sleep is essential for psychological well-being (Kalak et al., 2012; Brand et al., 2015).

Hence, this specific line of emerging researches, along with the extensive literature on the general relationship between sleep problems and psychiatric symptoms or disorders (Neylan et al., 1998; Pillar et al., 2000; Mellman and Hipolito, 2006; Spoormaker and Montgomery, 2008; Lauterbach et al., 2011; Van Der Kloet et al., 2013), has inspired and motivated us to further investigate the relationship between sleep disorders and psychiatric symptoms more closely and to further consider sleep problems as core factors of developing PTSD, anxiety and depression in order to design and implement new post-disaster psychiatric interventions for survivors who remained in temporary housing camps.

The present study had three research design features in an attempt to make scientific contributions to the existing literature. First, we deliberately chose *a special group* of earthquake survivors, i.e., those who still remained in temporary housing camps after the earthquake, as our primary research focus. Earthquake survivors are a heterogonous population rather than a homogenous one in their efforts to survive. For example, among those who live in temporary housing camps after an earthquake, some survivors will move back to their original places to rebuild their lives, others will immigrate to other places to seek new lives, and quite a few will still stay in temporary housing camps. We chose to focus on the earthquake survivors who had still stayed in temporary housing camps for about 2 years after the Wenchuan earthquake. One major reason for such a research decision is that most of these survivors were particularly vulnerable psychologically and physically as compared with their departed ones (Ma et al., 2013) and might need special psychological interventions to help them make a successful return to independent living. Second, we deliberately *took sleep problems* as our primary research focus and explored different dimensions of sleep quality in related to PTSD, anxiety, and depression. For those survivors who remained in temporary housing camps as a particularly vulnerable group, understanding, and addressing their daily sleep problems could be an innovative intervention strategy to use one stone to kill two birds, if certain relationships between sleep disorders and psychiatric symptoms can be found. Third, we *collected the data* in a way almost like an anthropology field work, finding and interviewing sleepless survivor, one after another, in various temporary houses, one after another, across various temporary camps, for more than 2 years. This process allowed us to collect the quality data to address our research goals as much as possible, while it did not permit for several other design options, such as a typical prevalence assessment (due to our focus on remaining survivors who have sleeping problems rather than each and every survivor across the camps), a typical longitudinal design (due to our two-year time consuming intense data collection process) or a typical experiment vs. control-group design (due to various difficulties to track down those departed survivors after 2 years of the earthquake).

Specifically, the present study attempted to achieve two goals: (1) assess sleep problems as well as their PTSD, depression and anxiety among the Wenchuan earthquake survivors who remained in temporary housing camps and (2) examine the relationship between different dimensions of sleep quality and PTSD, depression, and anxiety among these survivors.

## Materials and methods

### Participants

Participants in our research were the earthquake survivors who remained in temporary housing camps mainly in Qingchuan county and Beichuan county of Mianyang City, which were most severely affected by the earthquake, and explicitly reported poor or unsatisfying sleep quality. The Chinese government and provincial authorities built a large number of temporary housing camps to shelter survivors after the May 2008 Wenchuan earthquake. The temporary housing camps offered basic housing but no immediate access to employment. The survivors left the temporary housing camps for a variety of reasons, for example, seeking employment, starting a business or living with family members in cities outside the immediate earthquake zone. However, quite a few survivors remained there over the course of time. This study investigated the survivors who continued to reside in the temporary housing camps 17–27 months after the earthquake. The present study was reviewed and approved by the Wenzhou Medical University Research Ethics Committee. All participants in the study gave and signed informed consent in a written form in accordance with the Declaration of Helsinki before the interview started.

### Procedure

From August 2009 to June 2010, 17–27 months after the earthquake, as part of the psychological relief program supported by the Institute of Psychology, Chinese Academy of Sciences, we organized and conducted a community-based mental health investigation among adults mainly in Beichuan county and Qingchuan county. We advertised the study via radio and TV broadcast, posters, short messages of cell phones, and leaflets to recruit poor-quality sleepers from one county to another. In our research group, there were trained clinical psychologists, psychiatrists, and psychotherapists. We utilized local hospitals and community centers as the interview sites to carry out face-to-face one-on-one structured interviews. The interview consisted of a brief questionnaire about their own information and multiple tests regarding sleep and mental health issues. Before administrating the interviews to the participants, we obtained informed consent and introduced the aim, and significance of the interview in detail. Then all participants who agreed to participate signed a written informed consent. For illiterate participants, the consent was read to each and family members who signed on their behalf. The duration of one interview was about 45 min.

### Assessment instruments

#### Questionnaire on socio-demographic data and medical history of earthquake survivors

This questionnaire consisted of three parts: (1) Demographic characteristics such as gender, age, education level, marital status and occupation. (2) Sleep quality including major symptoms, time of occurrence and causes of insomnia. In this part, participants needed to answer two questions, “Do you think you have sleep problems? Yes or No” and “What is your main sleep problem? Insomnia, snoring, or other?”

#### The pittsburgh sleep quality index (PSQI)

Sleep quality was assessed by using the PSQI (Chen et al., 2005) which evaluated various aspects of functioning and well-being so as to provide an overall impression of sleep quality. The Pittsburgh Sleep Quality Index (PSQI) was edited by Wang et al. (1999). It consists of 19 self-report items and five clinician-rated items, among which the 19th self-report item and all of the five clinician-rated items were not scored. The remaining 18 self-report items measure seven component scores: subjective sleep quality, sleep latency, sleep duration, habitual sleep efficiency, sleep disturbance, sleep medication use, and daytime dysfunction. Each dimension was scored on a scale from 0 to 3, with total scores summed by all of the seven dimensional scores, and with higher scores indicating greater sleep problems. The present sample had acceptable internal consistency (α = 0.81).

#### PTSD checklist-civilian version (PCL-C)

The PTSD Checklist-Civilian Version was based on the DSM-IV and was designed to assess people's PTSD symptoms in daily life (Wang et al., 1999). It consists of 17 items and includes three dimensions: re-experience, avoidance, and hyper arousal. In the current sample, the PCL-C had high internal consistency (α = 0.92), with inter-item consistencies of the three dimensions respectively 0.86, 0.80, and 0.85. Intensity of each symptom was scored on a scale from 1 to 5, with 1 indicating “not at all” and 5 indicating “extremely.” A higher score indicated a more intense impact on mental health. In our study, the score of 44 was used as a boundary value of PTSD (Zhao et al., 2009).

#### Self-rating depression scale (SDS)

The Self-rating Depression Scale (SDS), edited by Zung (1965), consists of 20 items. Each item was scored on a scale from 1 to 4, with “1”suggesting “none or occasionally,” and “4”suggesting “always the case.” The SDS had acceptable internal consistency (α = 0.87). The boundary values of depression were as follows: 0–52, normal; 53–62, mild; 63–72, moderate; above 73, severe (Chen et al., 2005).

#### Self-rating anxiety scale (SAS)

The Self-rating Anxiety Scale (SAS), edited by Zung (1971), consists of 20 items. Each item was scored on a scale from 1 to 4, with “1”indicating “little time” and “4”indicating “most of the time or all the time.” The SAS had acceptable internal consistency (α = 0.85). The boundary values of anxiety were as follows: 0–49, normal; 50–59, mild; 60–69, moderate; above 70, severe (Chen et al., 2005).

### Data analysis

Data was analyzed by using Statistical Package for Social Sciences ver. 13.0 software. Descriptive statistics were used to display and describe the participants' backgrounds. Pearson correlation analysis and multiple regressions were used to examine relationships between the scores of sleep quality and PTSD, depression, anxiety, and to explore the role of different dimensions of sleep quality in predicting PTSD, anxiety, and depression in the study.

## Results

The current sample consisted of 387 participants, with 174 males and 212 females. The ages of the participants ranged from 19 to 63 years old, with an average age of 41.10. Demographic data were collected on gender, ethnicity, education and marital status. For example, 74.68% of the participants were the ethnicity of Han, 72.87% were married, and 54.78% of the participants received no more than junior high school education (Table 1).

**Table 1:** **Socio-demographic of the study subjects (*N* = 387)**.

	***N***	**Percent (%)**
**Female**	212	54.78
**Ethnicity of Han**	289	74.68
**Education**		
<High school	212	54.78
High school	83	21.45
Some college	51	13.18
≥Bachelor degree	34	8.79
**Marital status**		
Married/cohabitating	282	72.87
Separated/widowed/divorced	46	11.89
Unmarried	53	13.70

Table 2 summarizes the percentages and types of sleep problems. 83.20% of participants reported having sleep problems, and 79.33% of them considered insomnia as the most common sleep problem. What's more, among them survivors with 26–50 year olds reported the most sleep problems.

**Table 2:** **Percentages and types of symptoms of sleep problems**.

		**Percentage of sleep problems**
**Reporting yes**		83.20% (*n* = 322)
Sex	Males	42.55% (*n* = 137)
	Females	57.14% (*n* = 184)
Age	19–25 years	6.52% (*n* = 21)
	26–50 years	58.39% (*n* = 188)
	51–63 years	31.06% (*n* = 100)
**Reporting no**		16.54% (*n* = 64)
**Missing reporting**		0.26% (*n* = 1)
**Type of sleep problems**	
Insomnia (Sleep Latency, Difficulty in Staying Asleep, Early Awakening)		79.33% (*n* = 307)
Snore		3.62% (*n* = 14)
Others (e.g., Dreaminess)		7.75% (*n* = 30)
Missing		9.30% (*n* = 36)

Table 3 shows the Means and SDs of PLC-C, SDS, and SAS. Table 4 summarizes the prevalences of PTSD, depression and anxiety among survivors. A total of 47 cases were detected and the rate of PTSD was 12.14%. 36.43% of participants were experiencing depression, and 38.24% of participants were diagnosed with anxiety.

**Table 3:** **Descriptive statistics of PTSD, depression, and anxiety in survivors**.

	**Min**.	**Max**.	**Mean**	***S.D.***
PLC-C	17.00	73.00	32.65	11.41
SAS	25.00	83.00	45.80	12.61
SDS	25.00	77.00	48.53	11.89

**Table 4:** **Numbers and percentages of survivors in PTSD, SDS, and SAS**.

	**Number**			**Percentage (%)**		
	**PTSD**	**SDS**	**SAS**	**PTSD**	**SDS**	**SAS**
**Normal**	340	246	239	87.86	63.57	61.76
**Abnormal**	47	141	148	12.14	36.43	38.24
Mild		84	85		21.71	21.96
Moderate		51	50		13.18	12.92
Severe		6	13		1.55	3.16
Total		387			100.00	

Pearson correlation test, as shown in Table 5, the total scores of the PTSD Checklist-Civilian Version (PCL-C), the Self-rating Depression Scale (SDS), and the Self-rating Anxiety Scale (SAS) were significantly related to the Pittsburgh Sleep Quality Index (PSQI), *r* = 0.56, 0.60, 0.66, respectively. Furthermore, three psychological correlates, PCL-C, SDS, and SAS were also significantly correlated to the scores of the seven dimensions of the PSQI with the exception of the sleep medication use, all *p* s of each *r* s were reaching 0.01.

**Table 5:** **Descriptive statistics and zero-order correlations for all measures for PCL-C, SDS, SAS, and PSQI Scores**.

	**1**	**2**	**3**	**4**	**5**	**6**	**7**	**8**	**9**	**10**	**11**
1. PCL-C	−										
2. SDS	0.81^**^	−									
3. SAS	0.75^**^	0.71^**^	−								
4. PSQI	0.56^**^	0.60^**^	0.66^**^	−							
5. PSQI_lat	0.33^**^	0.34^**^	0.42^**^	0.75^**^	−						
6. PSQI_dur	0.42^**^	0.42^**^	0.45^**^	0.84^**^	0.57^**^	−					
7. PSQI_eff	0.42^**^	0.46^**^	0.48^**^	0.84^**^	0.60^**^	0.76^**^	−				
8. PSQI_dis	0.58^**^	0.61^**^	0.70^**^	0.59^**^	0.32^**^	0.40^**^	0.38^**^	−			
9. PSQI_med	0.28^**^	0.27^**^	0.32^**^	0.38^**^	0.19^**^	0.15^**^	0.17^**^	0.20^**^	−		
10. PSQI_dys	0.35^**^	0.42^**^	0.47^**^	0.69^**^	0.44^**^	0.43^**^	0.43^**^	0.33^**^	0.20^**^	−	
11. PSQI_sub	0.41^**^	0.42^**^	0.48^**^	0.76^**^	0.52^**^	0.59^**^	0.55^**^	0.39^**^	0.19^**^	0.57^**^	−
Mean	32.65	45.80	48.53	1.77	2.42	1.99	2.08	1.42	0.22	2.18	2.10
*SD*	11.41	12.61	11.89	0.62	0.80	1.16	1.17	0.66	0.65	0.91	0.70

PSQI, the aggregate score of The Pittsburgh Sleep Quality Index; PSQI_ lat, the score of the sleep latency component of PSQI; PSQI_dur, the score of the sleep duration component of PSQI; PSQI_ eff, the score of the sleep efficiency component of PSQI; PSQI_dis, the score of the sleep disturbance component of PSQI; PSQI_ med, the score of use of sleep medication component of PSQI; PSQI_dys, the score of daytime dysfunction component of PSQI; PCL-C, the aggregate score of the PTSD Checklist-Civilian Version; SDS, the aggregate score of the Self-rating Depression Scale; SAS, the aggregate score of the Self-rating Anxiety Scale. ^*^ p < 0.05,

** *p < 0.01*,

^***^p < 0.001, the same below.

The multiple regressions model in Table 6 shows that subjective sleep quality, sleep disturbance and sleep medication use significantly predicted PTSD, and explained totally 44.21% of the variances in PTSD, *F*_(11, 343)_ = 24.68, *p* < 0.001, *R*^2^ = 44.21%, the magnitude is medium (see Durlak, 2009). Habitual sleep efficiency, sleep disturbance, sleep medication use, and daytime dysfunction were significant predictors of depression and explained totally 46.73% of the variances in depression, *F*_(11, 343)_ = 27.33, *p* < 0.001, *R*^2^ = 46.73%, the magnitude is medium (see Durlak, 2009). Analysis showed that sleep disturbance, sleep medication use, and daytime dysfunction significantly predicted anxiety and explained totally 60.04% of the variances in anxiety, *F*_(11, 343)_ = 46.70, *p* < 0.001, *R*^2^ = 60.04%, the magnitude is large (see Durlak, 2009).

**Table 6:** **Regression analysis for PTSD, depression, and anxiety**.

	**PTSD**				**Depression**				**Anxiety**			
	***B***	***Std. Error***	**β**	***t***	***B***	***Std. Error***	**β**	***t***	***B***	***Std. Error***	**β**	***t***
Subjective sleep quality	1.80	0.92	0.11	1.96^*^	0.93	0.94	0.06	1.00	1.51	0.87	0.08	1.73
Sleep latency	−0.34	0.74	−0.02	−0.45	−0.61	0.76	−0.04	−0.80	0.76	0.70	0.05	1.08
Sleep duration	0.52	0.66	0.05	0.79	0.01	0.67	0.00	0.01	−0.07	0.62	−0.01	−0.11
Habitual sleep efficiency	0.66	0.65	0.07	1.01	1.74	0.67	0.18^**^	2.60	0.67	0.62	0.06	1.08
Sleep disturbance	6.71	0.83	0.39	8.04^**^	7.65	0.85	0.42^***^	9.00	9.37	0.79	0.48	11.85^***^
Usage of sleep medication	2.41	0.74	0.14	3.27^**^	2.02	0.75	0.11^**^	2.69	2.90	0.70	0.15	4.16^***^
Daytime dysfunction	1.03	0.64	0.08	1.61	2.10	0.65	0.16^***^	3.22	2.23	0.61	0.16	3.66^***^
*R^2^*	44.21%				46.73%				60.04%			
*F*	24.68^***^				27.33^***^				46.70^***^			

## Discussion

Strong researches on diverse psychiatric behavior, major psychiatric outcomes, and useful clinic treatment in Asian and Pacific Rim region have been published (Feng et al., 2011; Raphael and Ng, 2011; George et al., 2012; Ibrahim et al., 2013; Bae et al., 2015; Chee et al., 2015; Hong et al., 2016). The present study provided unique empirical evidence of sleep problems and psychiatric comorbidities which are associated with each other among Wenchuan Earthquake survivors who remained in temporary housing camps.

Major results revealed that12.1% of the earthquake survivors were found to have PTSD, 36.4% of them had depression, and 38.2% of them had anxiety. The percentages of PTSD in our study was the lowest compared with those in prior studies (i.e., 14.5% in Priebe et al., 2009 and 38.8% in Lee et al., 2009). This low PTSD rate could be due to: (1) the Chinese government and various communities providing timely and effective relief efforts after the earthquake, (2) potential cultural differences between the western countries and China in responding to the earthquake disaster, and (3) potential variations in using diagnostic tools and diagnostic times. The literature indicates that Asian earthquake survivors tend to express their stress with somatization. They tend to not think their physical symptoms are related to traumatic events (Goto and Wilson, 2003), and expressing personal feelings or admitting their own mental health problems to strangers is uncomfortable and shameful and thus they seldom seek help from psychiatrists or psychiatric services (Goto and Wilson, 2003; Kokai et al., 2004).

Further, 83.2% of the participants reported sleep problems and in those 79.3% reported their major symptom was insomnia. The rates of sleep problems in our study was the highest compared with the reported results in previous researches. For example, 55.0% of earthquake survivors had sleep disturbances 1 year after the 1999 Athens earthquakes and 60% of survivors complained of insomnia as the major symptom (Varela et al., 2008). This higher rate of sleeping problems could possibly be related to the following reasons: (1) the Chinese earthquake survivors had tendency to suppress their emotions more, which resulted in various symptoms of sleep disturbances, (2) the participants were voluntary in the present study and all of them reported that their sleep quality were poor or unsatisfying, and (3) Asian earthquake survivors were reported to tend to express their stress more with somatization (Goto and Wilson, 2003).

Furthermore, earthquake survivors' sleep quality was significantly predictive to developing PTSD, anxiety, and depression. Specifically, (1) sleep disturbance, sleep medication use, and subjective sleep quality were significantly related to PTSD; (2) habitual sleep efficiency, sleep disturbance, sleep medication use, and daytime dysfunction were significantly related to depression; and (3) sleep disturbance, sleep medication use, and daytime dysfunction were significantly related to anxiety. These new findings have clinic implications, calling for more attention to be paid to sleep disturbance and sleep problems that can accelerate psychiatric symptoms or disorders.

## Limitations and future research

Several limitations in this study should be noted. Firstly, we recruited subjects essentially by using convenience sampling which would affect the generalization of our results. Our samples were those earthquake survivors who were willing to participate in the study. These findings need to be further tested with those who had sleep problems but did not actively participate in the survey. Secondly, the study was essentially cross-sectional. Because of practical reasons, we were unable to follow up the participants. If we had a longitudinal dataset, we could have explored the casual relationship between variables effectively. Lastly, the longer the interval between the earthquake and the time-points of investigation, the more secondary or independent factors occur, that may also contribute to these comoties.

Despite these mentioned above, by design, our research focused on the particularly vulnerable group of earthquake survivors who still remained in the temporary housing camps after more than 2 years and documented in details their sleep problems in relating to their various psychological problems. Thus, it added new knowledge of sleep problems as a core variable for the Chinese earthquake survivors who remained in temporary housing camps by providing new empirical evidence of the relationship between sleep problems and psychological correlates. As a result, it has practical implications for developing effective mental health practices in trauma settings for earthquake survivors.

## Ethical approval

This research received ethical approval from Wenzhou Medical University Research Ethics Committee.

## Author contributions

JH contributed to the conception of the study. PJ, CL, and TZ collected all data, SJ analyzed and wrote the first draft of the paper. ZY contributed to the first drafting of the paper. SJ and ZY contributed to the critical revision of the paper. All authors approved the final manuscript for publication.

### Conflict of interest statement

The authors declare that the research was conducted in the absence of any commercial or financial relationships that could be construed as a potential conflict of interest.
